# Supplementary material for: Comprehensive Annotation and Functional Exploration of MicroRNAs in Lettuce
Source: Front Plant Sci. 2021 Dec 24;12:781836. doi: 10.3389/fpls.2021.781836 (PMC8739914; doi:10.3389/fpls.2021.781836)
Supplement: Supplementary file 2 [file Data_Sheet_8.ZIP › GSM2230750.plot/Lsa-miR319f_Lsat_1_v5_gn_9_9761.1_336_TPlot.pdf]

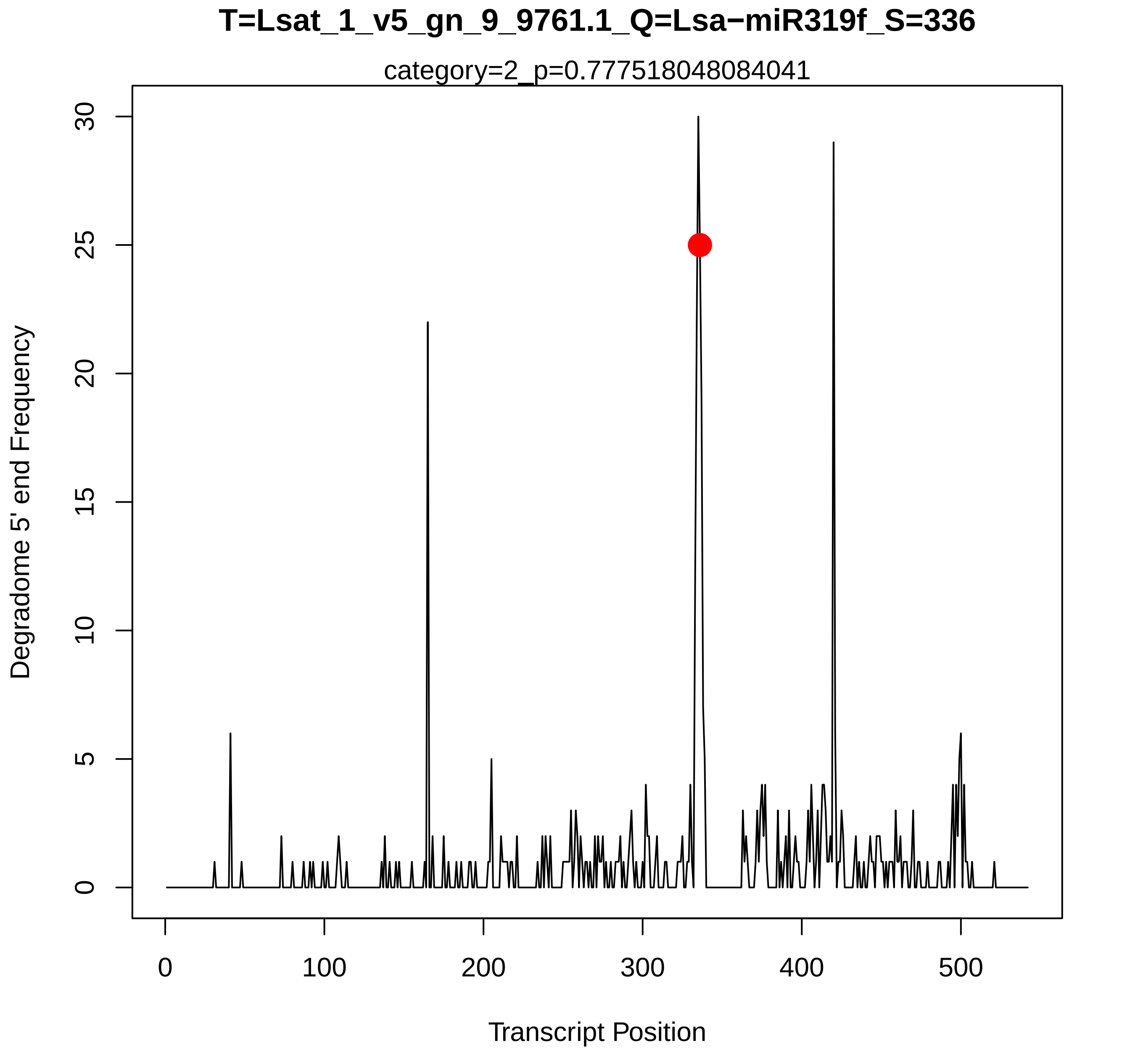
T=Lsat_1_v5_gn_9_9761.1_Q=Lsa−miR319f_S=336
category=2_p=0.777518048084041
Degradome 5' end Frequency
0
5
10
15
20
25
30
0
100
200
300
400
500
Transcript Position
